# Straightforward and robust synthesis of monodisperse surface-functionalized gold nanoclusters

**DOI:** 10.3762/bjnano.7.118

**Published:** 2016-09-08

**Authors:** Silvia Varela-Aramburu, Richard Wirth, Chian-Hui Lai, Guillermo Orts-Gil, Peter H Seeberger

**Affiliations:** 1 Max Planck Institute of Colloids and Interfaces, Department of Biomolecular Systems, Am Mühlenberg 1, 14476 Potsdam, Germany; 2 Freie Universität Berlin, Department of Biology, Chemistry, Pharmacy, Arnimallee 22, 14195 Berlin, Germany; 3 Helmholtz Centre Potsdam GFZ German Research Centre for Geosciences, Sec. 4.3, Telegrafenberg, 14473 Potsdam, Germany

**Keywords:** biomedical applications, carbohydrates, functionalization, gold nanoclusters, robust

## Abstract

Gold nanoclusters are small (1–3 nm) nanoparticles with a high surface area that are useful for biomedical studies and drug delivery. The synthesis of small, surface-functionalized gold nanoclusters is greatly dependent on the reaction conditions. Here, we describe a straightforward, efficient and robust room temperature one-pot synthesis of 2 nm gold nanoclusters using thioglucose as a reducing and stabilizing agent, which was discovered by serendipity. The resultant monodisperse gold nanoclusters are more stable than those generated using some other common methods. The carboxylic acid contained in the stabilizing agent on the cluster surface serves as anchor for nanocluster functionalization. Alternatively, the addition of thiols serves to functionalize the nanoclusters. The resulting non-cytotoxic nanoclusters are taken up by cells and constitute a tuneable platform for biomedical applications including drug delivery.

## Findings

Nanoparticles ranging in size from 1 to 100 nm are ideal tools to study biological processes [1–2]. Many different materials, including gold, have been used to create nanoparticles [3–6]. Gold nanoparticles are an attractive platform because of their biocompatibility, low toxicity, and low immunogenicity [7], their inherent optoelectronic properties [8] and high transmission electron microscopy (TEM) contrast. They are relatively easy to synthesize, functionalize, are biocompatible and have controllable optical properties [3,9–12]. Therefore, gold nanoparticles functionalized with carbohydrates [13], proteins [14], antibodies [15] and DNA [16] are commonly used as multivalent materials for biological studies. Gold nanoparticles have been used in vivo as radiotracers [15,17], for targeted delivery [18] and, when functionalized with carboxylic acids, inhibit β-amyloid fibril growth related to Alzheimer’s disease [19]. Gold nanoclusters (NCs) are gold nanoparticles ranging in size between 1 and 3 nm, with interesting physicochemical properties and increased surface area for drug delivery applications [20].

There are several methods to synthesize gold nanoparticles. In addition to the reduction of HAuCl_4_ with citrate at high temperatures [21], sodium borohydride can act as a reducing agent while an alkanethiol stabilizes the nanoparticles [22]. The latter method was used to prepare glyconanoparticles by adding thiol-terminated glycoconjugates [23]. Gold nanoparticles have also been prepared under reflux using 1-thioglucose as reducing and stabilizing agent [24] but the resulting nanoparticles are too unstable to be used as biosensors [25].

In an effort to create monodisperse, stable and surface-functionalized gold nanoclusters, we explored 1-thioglucose as a stabilizing and reducing agent. By serendipity we discovered a novel one-pot method to prepare gold nanoclusters using 1-thioglucose at room temperature. This simple and robust synthesis produces stable, and monodisperse nanoclusters. Oxidation of the carbohydrate results in carboxylic acid as determined by X-ray photoelectron spectroscopy (XPS). Coupling to the carboxylic acids or addition of thiols functionalizes the NCs that are taken up by cells but are less cytotoxic than NCs prepared by other methods.

**Figure 1:**
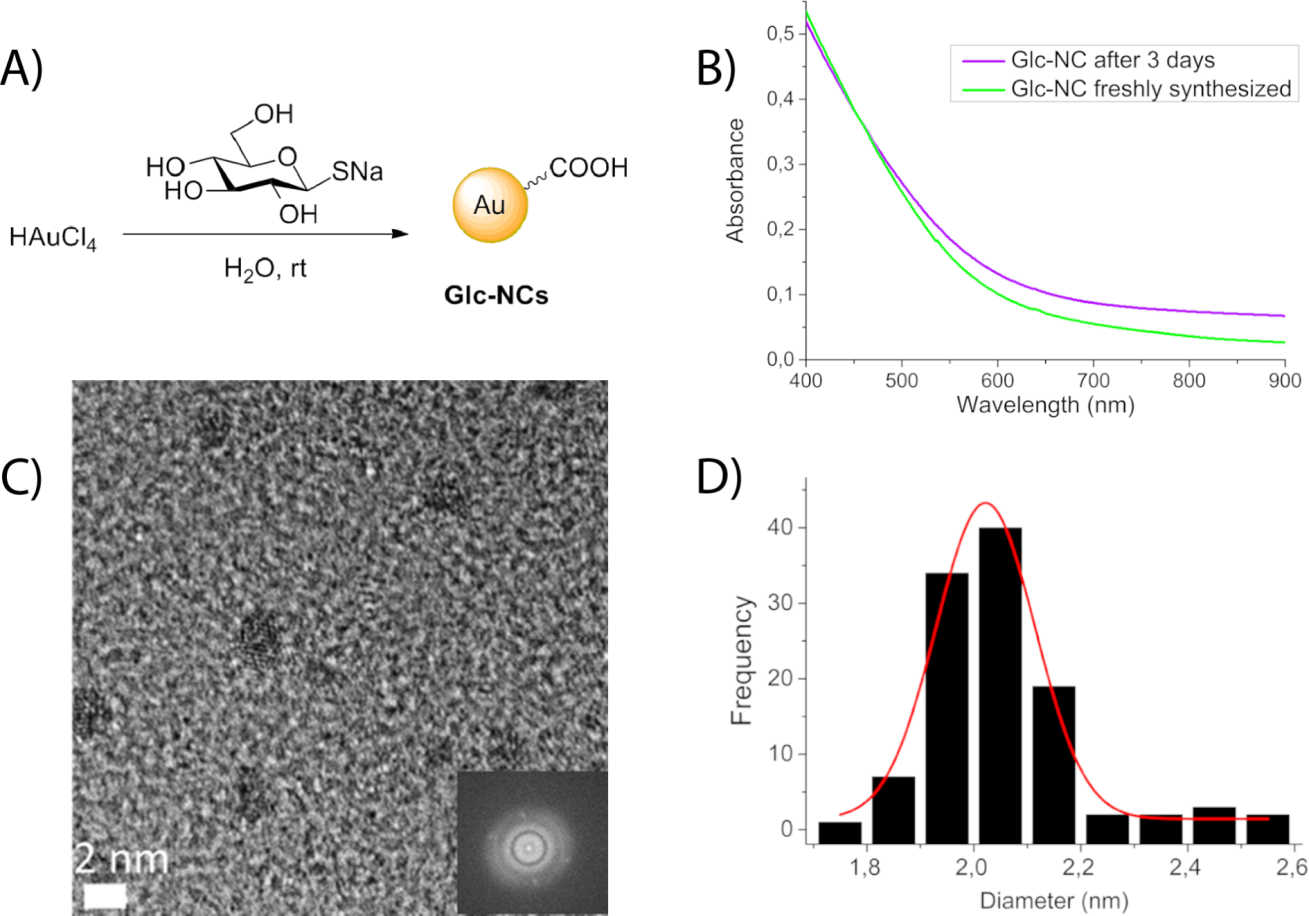
A) One-pot synthesis of **Glc-NCs** at room temperature; B) UV–vis spectra of freshly synthesized **Glc-NCs** and after three days are indicative of stable particles; C) high-resolution TEM bright-field image of **Glc-NCs** showing monodisperse nanoclusters, scale bar 2 nm; D) size distribution of 110 nanoclusters of **Glc-NCs** yielding diameters of 2.02 ± 0.18 nm.

During experiments exploring different methods for the synthesis of gold tetrapods [26], we found that simply the addition of 1-thioglucose as reducing agent to gold salts resulted in the formation of monodisperse gold NCs (Figure 1A). The reaction produced the same products at any temperature between 0 and 90 °C and thereby stood in stark contrast to all known literature procedures [27–29] that were sensitive to all reaction conditions including the speed of the stirrer. The influence of the gold to 1-thioglucose ratio on the yield and quality of the glucose-stabilized gold nanoclusters (**Glc-NCs**) was determined (Table S1, Supporting Information File 1). The nanoclusters aggregated within 5 h at very high ratios of gold ions to 1-thioglucose (Figure S1, Supporting Information File 1). At higher gold ion concentrations, NCs that are not fully coated with stabilizer aggregate. At high thioglucose concentrations, NCs do not form. Monodisperse gold NCs (2.02 ± 0.18 nm) were obtained as determined by high resolution TEM (Figure 1C,D) and dynamic light scattering (DLS) (Figure S4, Supporting Information File 1). The one-pot synthesis of **Glc-NCs** is independent on the temperature between 0 and 90 °C (Figure S5, Supporting Information File 1).

The **Glc-NCs** are more stable than nanoclusters that were stabilized with cetyltrimethylammonium bromide (**CTAB-NCs**) and tetrakis(hydroxymethyl)phosphonium chloride (**THPC-NCs**) as determined by UV absorbance. Gold nanoparticles smaller than 5 nm do not display a plasmon band. The **Glc-NCs** are stable and showed the same absorbance profile after three days of dialysis (Figure 1B). In contrast, the **CTAB-NCs** and the **THPC-NCs** exhibit plasmon bands (Figure S6, Supporting Information File 1). The **CTAB-NCs** showed a plasmon band directly after synthesis, which shifted to longer wavelengths after three days, suggesting an increasing rate of aggregation. TEM images of freshly prepared **CTAB-NCs** revealed polydisperse nanoclusters in terms of both size and shape (data not shown). The absorbance profile of **THPC-NCs** did not show a plasmon band after the synthesis. However, after three days, a plasmon band appeared, revealing particle aggregation. The surface charge did not change upon dialysis for any sample as indicated by the zeta potential (Figure S7, Supporting Information File 1).

**Figure 2:**
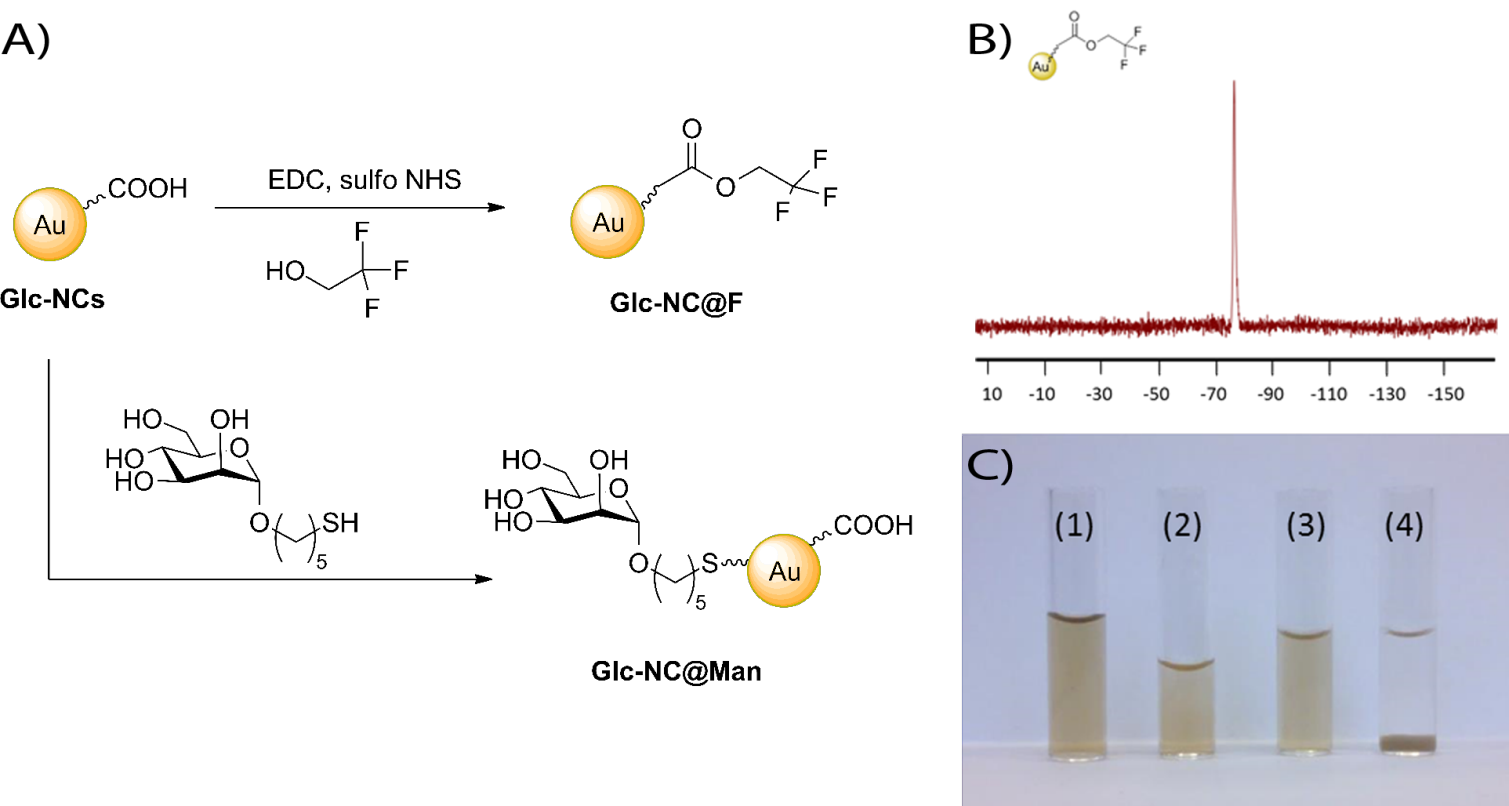
A) Functionalization of **Glc-NCs** by coupling trifluoroethanol to the carboxylic acid groups on the surface of the nanocluster to yield **Glc-NC@F** and via thio-substitution of pentylthiol mannoside on the gold surface giving **Glc-NC@Man**; B) ^19^F NMR of **Glc-NC@F** after dialysis showing one product peak; C) vials containing (1) **Glc-NCs** (2) **Glc-NC@Man** (3) **Glc-NCs** + ConA (4) **Glc-NC@Man** + ConA. Aggregation is only observed upon addition of the lectin ConA (4).

To better understand the role of the stabilizing agent used to prepare **Glc-NCs**, XPS was employed (Figure S8, Supporting Information File 1). The gold core of the nanoclusters (confirmed by the Au 4f scan) is stabilized through an Au–S bond (162.5 eV, S 2p scan). C–O bonds and a carbonyl group were detected on the nanocluster surface by C 1s (286 eV and 288.5 eV, respectively) and O 1s (531.5 eV and 533 eV, respectively) scans (Table S2 and Table S3, Supporting Information File 1). The carbonyl signal likely is indicative of the oxidation of hydroxy groups of the carbohydrate as the precursor 1-thio-glucose contains no carbonyl group. The carboxylic acid in the stabilizing agent was confirmed by the XPS O–C=O signal and IR bands at 3270, 1732 and 1014 cm^−1^ (Figure S9, Supporting Information File 1). To confirm the XPS and IR analyses, trifluoroethanol was coupled to form **Glc-NC@F** to be monitored by using ^19^F NMR (Figure 2A,B). The ^19^F NMR signal of the starting material shifts after 30 min to show both starting material and product peaks (Figure S10a, Supporting Information File 1). Only the product peak remained after dialysis. Twenty stabilizer molecules per nanocluster were measured by comparing the number of trifluoroethanol molecules coupled to **Glc-NCs** using an internal standard (Figure S10b, Supporting Information File 1). Thus, we demonstrated that **Glc-NCs** can be functionalized via coupling to carboxylic acid groups.

To illustrate the surface functionalization of **Glc-NCs**, pentylthiol mannoside (Scheme S1, Supporting Information File 1) was incubated with **Glc-NCs** overnight to yield **Glc-NC@Man** (Figure 2A). After dialysis, the IR spectrum revealed two new peaks corresponding to the C–H bonds of the linker at 2856 and 2925 cm^−1^. Apparently, all carbohydrates reacted as no S–H peaks were observed (Figure S11, Supporting Information File 1). Functional evidence for the formation of **Glc-NC@Man** was obtained by aggregating **Glc-NC@Man** with the addition of the mannose-binding lectin concanavalin A (ConA). Unfunctionalized **Glc-NCs** fail to aggregate since oxidized thio-glucose is not recognized by ConA (Figure 2C). **Glc-NC@Man** are monodisperse prior to aggregation by the addition of ConA as judged by TEM (Figure S12, Supporting Information File 1).

**Figure 3:**
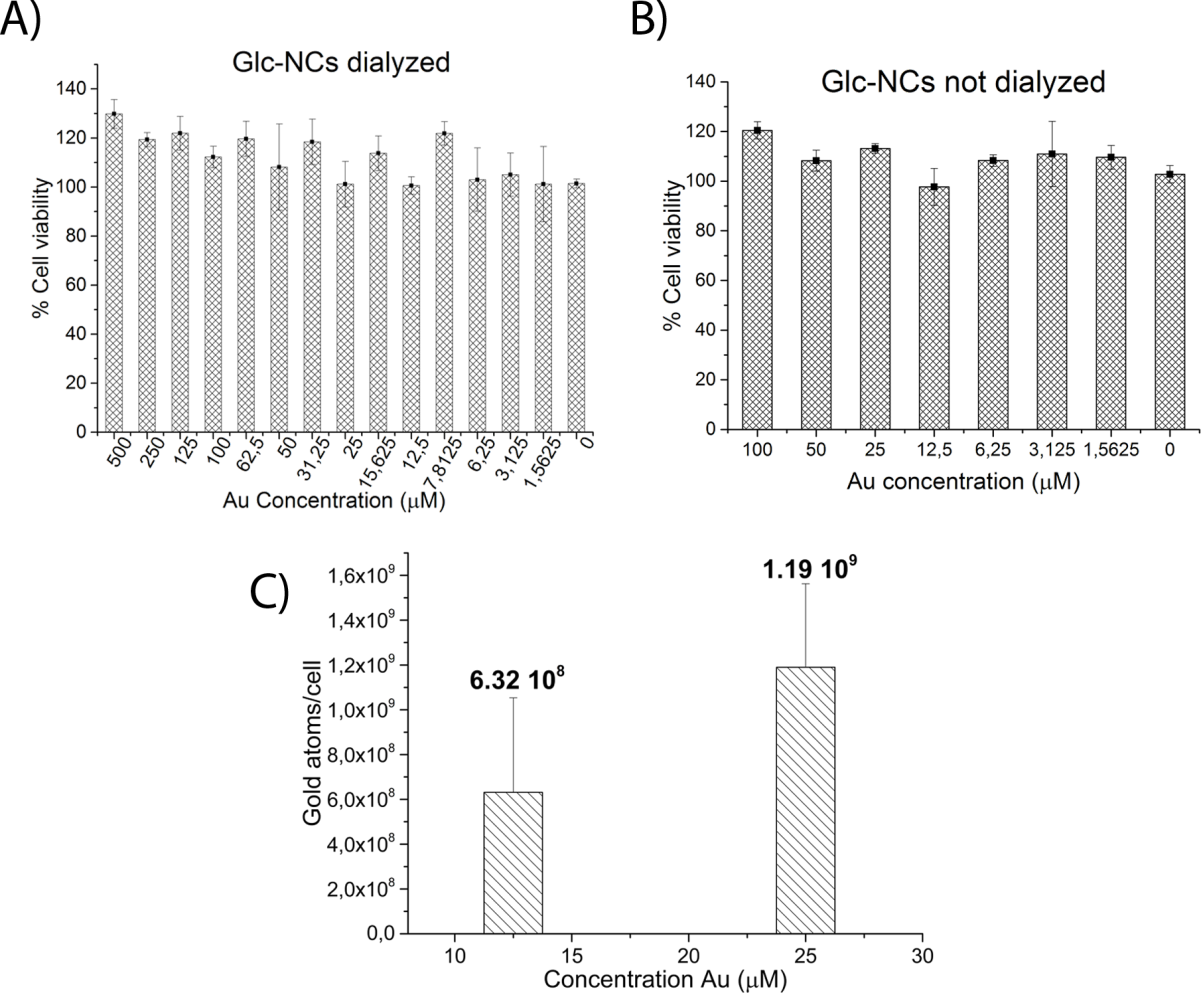
Cell viability of **Glc-NCs** A) purified and B) without purification incubated for one day with L929 cells. The **Glc-NCs** were not toxic at any of the concentrations studied. C) Cellular uptake of **Glc-NCs** when incubated with L929 cells for one day. Gold concentration taken up by the cells was measured with ICP-OES and showed an uptake of 10^8^–10^9^ gold atoms per cell.

Nanocluster cytotoxicity was assessed by incubating the nanoclusters for one day with the mouse cell line L929 for a proof-of-principle study. Cell viability was measured using the MTS [3-(4,5-dimethylthiazol-2-yl)-5-(3-carboxymethoxyphenyl)-2-(4-sulfophenyl)-2*H*-tetrazolium inner salt] assay [30]. The cytotoxicity of **Glc-NCs**, **CTAB-NCs** and **THPC-NCs** was compared. **CTAB-NCs** were toxic even at low concentrations (0.2–25 µM), whereas both **THPC-NCs** (Figure S13, Supporting Information File 1) and **Glc-NCs** did not show any toxicity at 500 µM (Figure 3A). To test whether the free stabilizers were affecting the cytotoxicity, both **Glc-NCs** and **THPC-NCs** were measured after synthesis without dialysis. **Glc-NCs** were not cytotoxic at any concentration tested (Figure 3B), whereas **THPC-NCs** were toxic at 100 µM (Figure S14, Supporting Information File 1), indicating that **Glc-NCs** are suitable for biological experiments even without purification.

Cellular uptake of the nanoclusters was studied by incubating 12.5 and 25 µM **Glc-NCs** with the same cell line. After 24 h, the cells were washed with buffer and then treated with aqua regia to transform the gold nuclei into gold ions that were detected by an inductively coupled plasma optical emission spectrometer (ICP-OES). The **Glc-NCs** are cell permeable as 10^8^–10^9^ gold atoms were delivered per cell and can potentially be used in cellular delivery applications (Figure 3C).

In summary, we developed a straightforward, robust and efficient one-pot method to prepare glucose-stabilized gold nanoclusters (**Glc-NCs**). The resulting 2 nm nanoclusters are monodisperse and more stable than gold nanoclusters synthesized by other methods. Functionalization of the **Glc-NCs** is achieved either via coupling to the carboxylic acid of the stabilizing agent or substitution with a thio-functionalized molecule. **Glc-NCs** are non-toxic, but are taken up by L929 cells. Surface functionalization of **Glc-NCs** with biomolecules opens opportunities for drug delivery applications.

## Supporting Information

File 1 Additional experimental data.
