# Supplementary material for: Straightforward and robust synthesis of monodisperse surface-functionalized gold nanoclusters
Source: Beilstein J Nanotechnol. 2016 Sep 8;7:1278–83. doi: 10.3762/bjnano.7.118 (PMC5082343; doi:10.3762/bjnano.7.118)
Supplement: File 1 — Additional experimental data. [file Beilstein_J_Nanotechnol-07-1278-s001.pdf]

Supporting Information

for

Straightforward and robust synthesis of monodisperse surface-functionalized gold nanoclusters

Silvia Varela-Aramburu[1,2], Richard Wirth[3], Chian-Hui Lai[1], Guillermo Orts-Gil[1] and Peter H. Seeberger*[1,2]

Address: [1]Max Planck Institute of Colloids and Interfaces, Department of Biomolecular Systems, Am Mühlenberg 1, 14476 Potsdam, Germany; [2]Freie Universität Berlin, Department of Biology, Chemistry, Pharmacy, Arnimallee 22, 14195 Berlin, Germany and [3]Helmholtz Centre Potsdam GFZ German Research Centre for Geosciences, Sec. 4.3, Telegrafenberg, 14473 Potsdam, Germany

Email: Peter H. Seeberger* - peter.seeberger@mpikg.mpg.de

* Corresponding author

Additional experimental data

Table of Contents

Synthesis and Characterization of Gold Nanoclusters 3
Synthesis of glucose-based gold nanoclusters (Glc-NCs) 3
Synthesis of THPC-based gold nanoclusters (THPC-NCs) 4
Synthesis of CTAB-based gold nanoclusters (CTAB-NCs)............................ 4
Transmission electron microscopy.. 5

UV–vis spectrometry 6

Zeta potential 6

XPS measurements 7

Infrared spectrometry 9

Functionalization and Characterization of Glc-NCs 9

Synthesis of Glc-NC@F 9

Synthesis of mannose with thio linker. 11

Synthesis of Glc-NC@Man 13

Biological Studies 15

Cell culture 15

MTS Assay 15

ICP-OES 16

References 17

Synthesis and Characterization of Gold Nanoclusters

Synthesis of glucose-based gold nanoclusters (Glc-NCs)

In a representative synthesis, 1-thio-β-D-glucose sodium (Glc-SNa) (80 µL, 41.2 mM) was added to $HAuCl_4$ (1 mL, 2.89 mM) at room temperature. In a few seconds a change in color from yellow to brownish was observed indicating the formation of the gold nanoclusters. The solution was dialyzed in two cycles of 1.5 L of ultrapure water.

Table S1: Experimental details for the synthesis of Glc-NCs by varying the ratio between the reactants.

**Entry**	**Glc-SNa (41.2 mM)**	**$HAuCl_4$ (2.9 mM)**	**Ratio Gold:Glc**	**Observations**
**1**	6.68 µL	500 µL	1:0.2	Light brown (unstable)
**2**	16.71 µL	500 µL	1:0.5	Dark-brown (unstable)
**3**	33.41 µL	500 µL	1:0.9	Brown
**4**	50.13 µL	500 µL	1:1.4	Brown
**5**	66.83 µL	500 µL	1:1.9	Light brown
**6**	83.13 µL	500 µL	1:2.4	White precipitate
**7**	100.26 µL	500 µL	1:2.8	Nothing happens

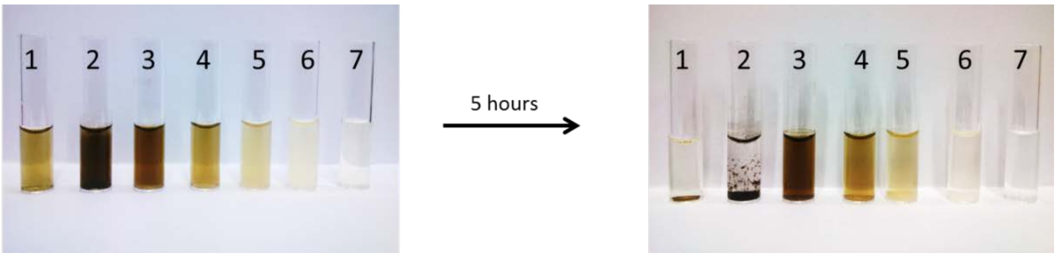

Figure S1: Glc-NCs freshly synthesized as shown in Table S1 and after 5 h.

Synthesis of THPC-based gold nanoclusters (THPC-NCs)

As described previously [1], a solution of NaOH (1.5 mL, 0.2 M) was added to 45.5 mL of ultrapure water and stirred with the reducing agent tetrakis(hydroxymethyl)phosphonium chloride (THPC, 1 mL of a solution of 0.3 mL of 80% aqueous solution diluted to 25 mL with ultrapure water). The mixture was stirred for five minutes before $HAuCl_4$ was added (2 mL, 25 mM). The solution turned to a brown-orange color. The nanoclusters were further purified by two cycles of dialysis against 1.5 L of ultrapure water (Figure S2).

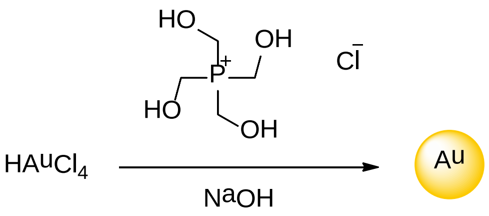

Figure S2: Synthetic reaction of THPC-NCs.

Synthesis of CTAB-based gold nanoclusters (CTAB-NCs)

In a representative synthesis, 10 mL of a solution of gold nanoclusters, $HAuCl_4$ (250 nM) was mixed with cetyltrimethylammonium bromide (CTAB, 75 mM) and afterwards reduced by $NaBH_4$ (600 nM). The reaction mixture was stirred for five minutes allowing the escape of the gas formed during the reaction. The nanoclusters were purified by two cycles of dialysis against 1.5 L of ultrapure water (Figure S3).

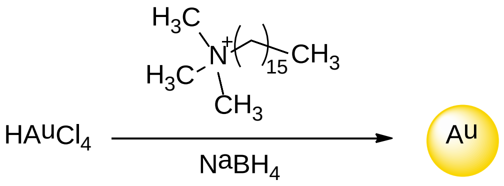

Figure S3: Synthetic reaction of CTAB-NCs.

Transmission electron microscopy

TEM measurements were performed on a Zeiss EM 912 Omega. The samples were prepared by immersion of grids into a small volume of the sample and subsequent solvent evaporation in a dust protected atmosphere.

High resolution TEM

High-resolution imaging was performed using a Tecnai F20 X-Twin transmission electron microscope at GFZ Potsdam. The TEM is equipped with a field emission gun as electron emitter. TEM bright-field images were acquired as energy filtered images using a Gatan imaging filter GIF. A 20 eV window was applied to the zero-loss peak. Data were evaluated with the Gatan Digital Micrograph software package.

Dynamic light scattering

DLS measurements were carried out at a scattering angle of 173° with a Malvern Zeta Nanosizer working at 4-mW He–Ne laser (633 nm). The **Glc-NCs** were measured in milliQ water (Figure S4).

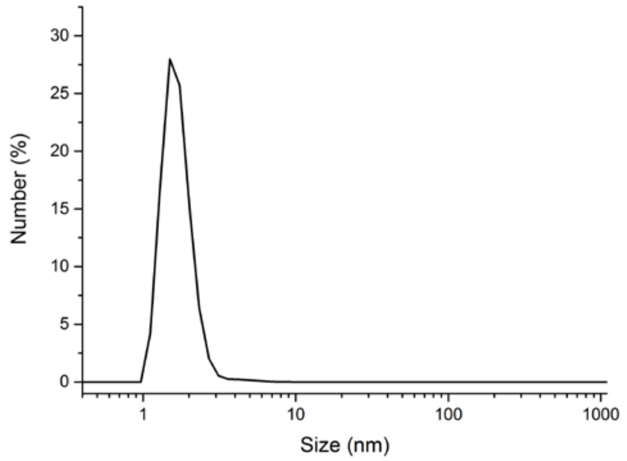

Figure S4: Dynamic light scattering measurement of hydrodynamic diameter of Glc-NCs in MilliQ water.

UV–vis spectrometry

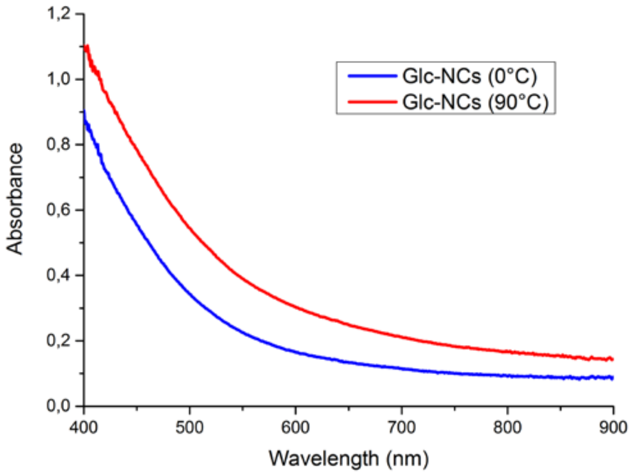

Figure S5: UV–vis spectra of Glc-NCs when synthesized at 0°C and 90°C.

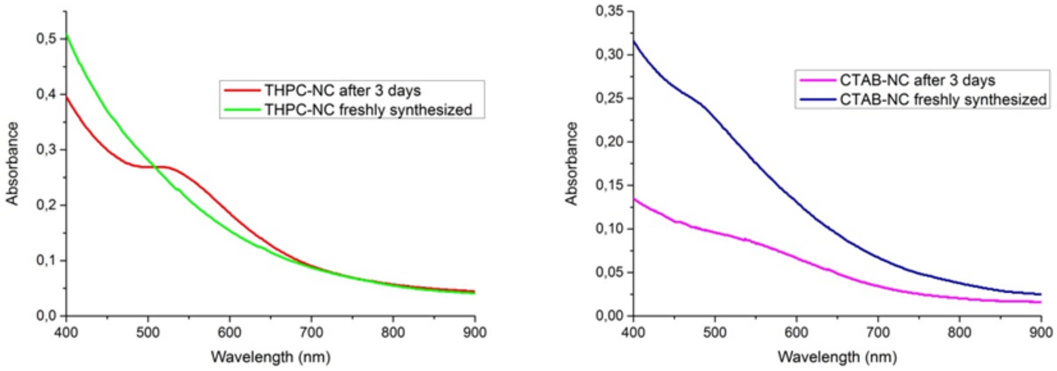

Figure S6: Ultraviolet spectra of (a) **THPC-NCs** and (b) **CTAB-NCs**, freshly synthesized and after 3 days.

Zeta potential

A Malvern Zetasizer instrument was used to measure the electrophoretic mobility of nanoparticles at different times of dialysis against MilliQ water. The Helmholtz-Smoluchowski equation was used to correlate the measured electrophoretic mobilities to the zeta potentials. Three replicates of each sample were measured six times at 25 °C in MilliQ water.

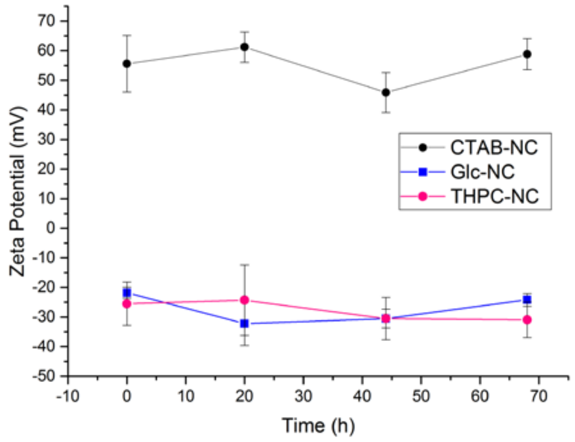

Figure S7: Zeta potential measurement of Glc-NC, THPC-NC and CTAB-NC depending on the time of dialysis against MilliQ water.

XPS measurements

X-ray photoelectron spectroscopy (XPS) measurements were carried out with a Thermo Scientific K-Alpha X-ray Photoelectron Spectrometer. A monochromatic Al Kα radiation (hν = 1486.6 eV) was used. A 200 eV analyzer pass energy and a 1 eV energy step size were used in survey scan measurements. Element scans were performed with a 50 eV analyzer pass energy and a 0.1 eV energy step size. All the obtained binding energies were referenced to carbon 1s peak at 284.8 eV. The samples were prepared on gold thin film substrates.

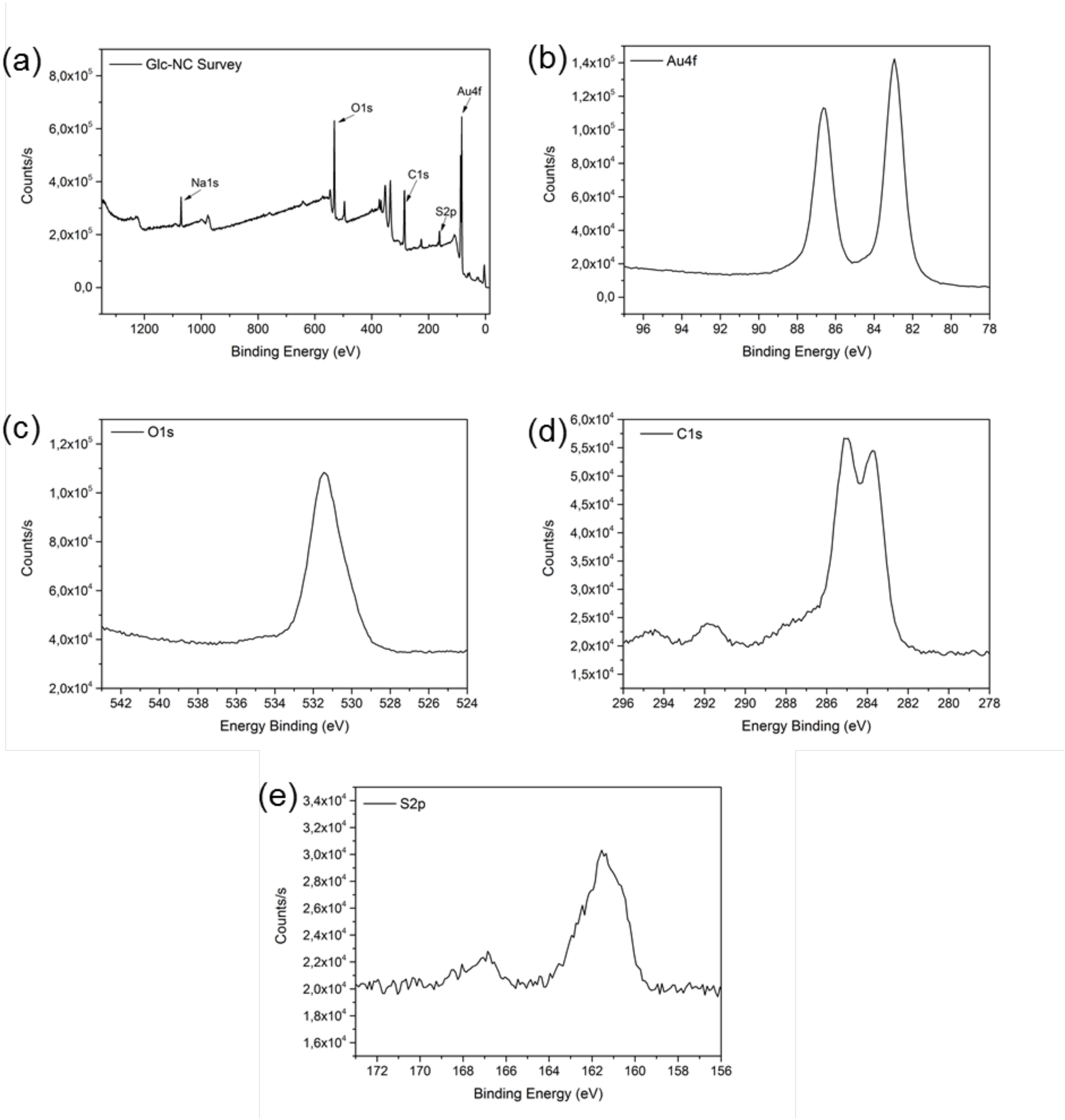

Figure S8: XPS data of **Glc-NCs**. (a) Survey scan of **Glc-NCs**. (b) Au4f, (c) O1s, (d) C1s, (e) S2p, elemental scans of **Glc-NCs**.

Table S2: XPS data of Glc-NCs**.**

**Element**	**Peak BE**	**FWHM eV**	**Area (P) counts/s.eV**
**C1s**	284.8	3.29	802670.4
**O1s**	531.59	3.02	1131512
**S2p**	161.78	1.78	161929.7
**Au4f**	83.52	2.82	2395402

Table S3: XPS analysis of Glc-NC by chemical state.

Chemical state	Peak BE	Chemical state	Peak BE	Chemical state	Peak BE
**C-C**	284.8	**C-O**	531.5	**Au-S**	162.5
**C-O-C**	286	**C=O**	533		
**O-C=O**	288.5				

Infrared spectrometry

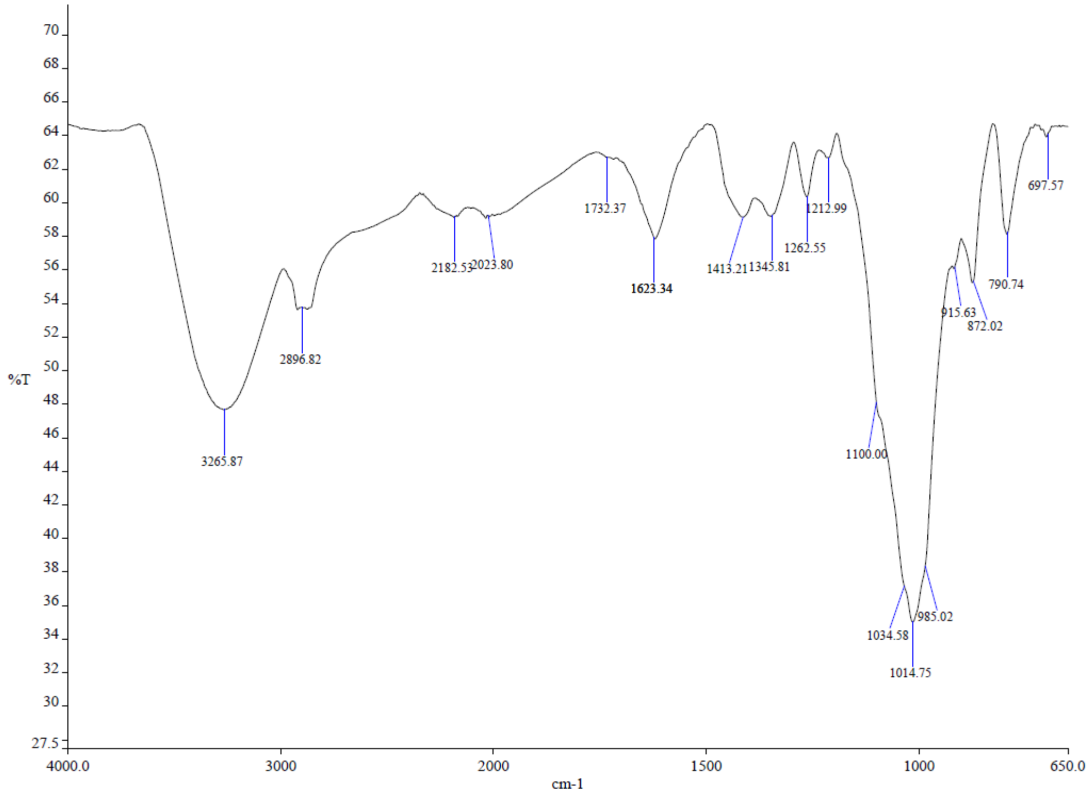

Figure S9: IR spectrum of **Glc-NCs**.

Functionalization and Characterization of Glc-NCs

Synthesis of Glc-NC@F

In a representative synthesis, a solution of **Glc-NCs** (1 mL, 0.358 µmol) is mixed with 1 mL of PBS. The resulting solution is diluted with MilliQ water to 10 mL and then 1-ethyl-3-(3-dimethylaminopropyl)carbodiimide (1.373 mg, 7.16 µmol) and *N*-

hydroxysulfosuccinimide (1.555 mg, 7.16 µmol) were added under stirring and let react for five minutes. 2,2,2-trifluoroethanol (5.15 µl, 0.072 mmol) were then added and let react under stirring for 2 h. The **Glc-NC@F** was purified against 1.5 L MilliQ water twice.

^{19}F NMR of Glc-NC@F

To calculate the number of -CF_3 groups coupled to **Glc-NCs**, CsF was used as an internal standard (IS). For that, 2.7 µmol were added to the NMR tube containing the purified product and ^{19}F NMR was measured. The integration of the peaks gave the ratio of each component in the NMR tube. The integration IS:-CF_3 was 1:0.13.

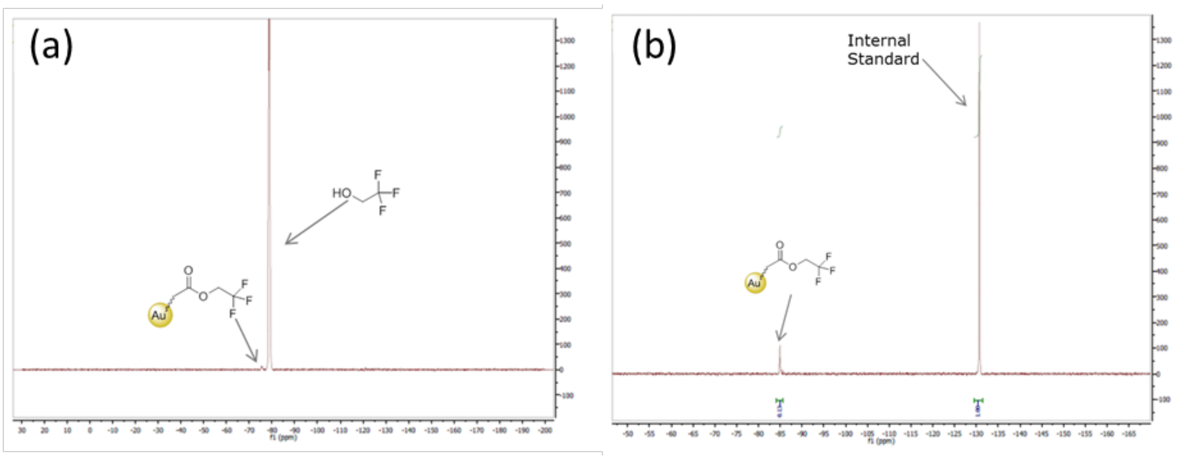

Figure S10: (a) ^{19}F-NMR spectrum after 5 minutes of reaction showing one peak of the starting material and a small peak of **Glc-NC@F**. (b) ^{19}F-NMR spectrum of **Glc-NC@F** after purification containing CsF as IS.

The exact gold concentration was measured with Inductively Coupled Plasma Optical Emission Spectrometer (ICP – OES) (Optima 8000; Perkin Elmer¸ Massachusetts; USA). For that an external calibration series from 0.1 mg/L to 5 mg/L was prepared using a gold standard solution.

Table S4: Concentration of both gold and $-CF_3$ in the NMR tube.

NMR Tube	Gold	$-CF_3$
**Concentration**	832 nmol	117 nmol
**Number of atoms/molecules**	$5\cdot10^{17}$ atoms	$7.046\cdot10^{16}$ molecules

To calculate the number of $-CF_3$ per nanocluster, the nanocluster was considered a sphere with diameter 2 nm and density ρ = 19.3 g/cm^3, using equation (1).

$$m = \frac{4}{3}\pi r^3 \rho \qquad (1)$$

The nanocluster mass obtained was $4.55\cdot10^{-20}$ g which is 139 atoms per nanocluster, giving 20 $-CF_3$/nanocluster.

Synthesis of mannose with thio linker

To functionalize Glc-NC, a mannose with a thio linker was synthesized (**3**). For this synthesis the peracetylated mannose **1** was coupled with 5-chloropentanol in presence of $BF_3.OEt_2$ to the anomeric position of the acetyl protected carbohydrate and via a S_N2 reaction, the chloride was converted to acetylated thiol **2** [2]. The hydrolysis of the acetylated groups in basic conditions yield **3** [3].

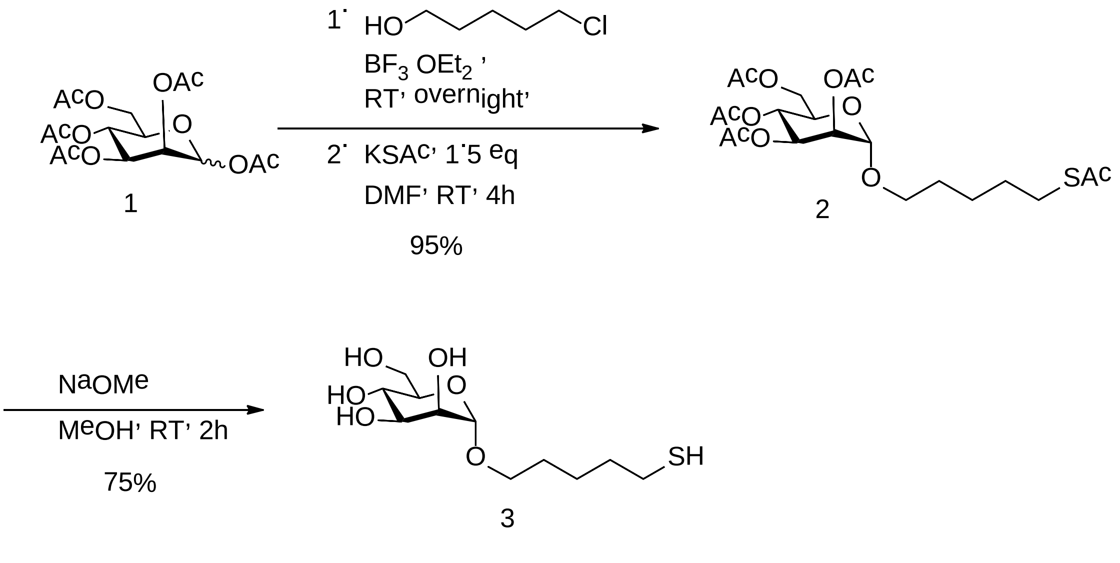

Scheme S1: Synthesis of mannose with thio linker.

(2R,3R,4S,5S,6S)-2-(acetoxymethyl)-6-((5-(acetylthio)pentyl)oxy)tetrahydro-2*H*-pyran-3,4,5-triyl triacetate (2)

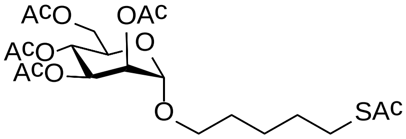

Compound **1** (4.33 g, 11.1 mmol) was dissolved in anhydrous CH_2Cl_2 and 5-chloropentan-1-ol (1.54 mL, 13.32 mmol) were added and stirred for one hour. Then the mixture was cooled to 0°C, $BF_3.OEt_2$ (6.43 mL, 51.2 mmol) was slowly added drop wise and continuously stirred for 12 hours at room temperature under an Argon atmosphere. The crude mixture was diluted with CH_2Cl_2 and water and the phases were separated. The organic layer was washed three times with saturated aqueous $NaHCO_3$ and NaCl. The organic layers were combined, dried over sodium sulfate, filtered and concentrated in vacuum. Since the compound was difficult to purify due to the hydrolysis, the next step was performed without further purification.

The previous compound (0.1 g, 0.221 mmol) was dissolved in anhydrous DMF and stirred with KSAc (0.50 g, 4.42 mmol) overnight at room temperature. The mixture was extracted with EtOAc and concentrated in vacuum to get compound **2** (0.1033 g, 95%). It was purified by flash chromatography (5→15→25% EtOAc/hexanes).

^{1}H NMR (400 MHz, $CDCl_3$) δ 5.32 (dd, 1H, H-3), 5.27 (d, 1H, H-4), 5.21 (dd, J = 3.3, 1.7 Hz, 1H, H-2), 4.78 (d, J = 1.4 Hz, 1H, H-1), 4.30 – 4.23 (m, 1H, H-6), 4.11 – 4.05 (m, 1H, H-6), 3.98 – 3.92 (m, 1H, H-5), 3.70 – 3.62 (m, 1H, CH_2-7), 3.46 – 3.39 (m, 1H, CH_2-7), 2.86 (t, J = 7.3 Hz, 2H, CH_2-11), 2.31 (s, 3H, CH_3-SAc), 2.14 (s, 3H, CH_3), 2.09 (s, 3H, CH_3), 2.03 (s, 3H, CH_3), 1.98 (s, 3H, CH_3), 1.63 – 1.54 (m, 4H CH_2-8,10), 1.46 – 1.37 (m, 2H, CH_2-9). LC MS. Calc for $[M+Na]^+$ 492.5 $C_{11}H_{21}N_3O_6$, found 492.4

(2R,3S,4S,5S,6S)-2-(Hydroxymethyl)-6-((5-mercaptopentyl)oxy)tetrahydro-2*H*-pyran-3,4,5-triol (3)

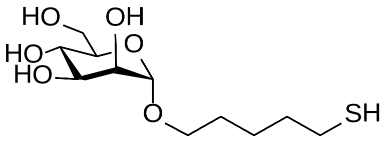

Compound **2** (50 mg, 0.102 mmol) was dissolved in 1.015 mL of MeOH, then sodium methoxide (5.48 mg, 0.102 mmol) was added and stirred for two hours at room temperature. The mixture was neutralized with amberlite-IR120 (H+) resin, filtered and concentrated in vacuum to get compound **3**.

^{1}H NMR (400 MHz, CD_3OD) δ 4.71 (s, 1H), 3.80 (d, J = 9.8 Hz, 1H), 3.76 (s, 1H), 3.72 (d, J = 9.5 Hz, 1H), 3.70 – 3.66 (m, 1H), 3.65 (d, J = 3.5 Hz, 1H), 3.57 (t, J = 9.4 Hz, 1H), 3.51 (d, J = 8.2 Hz, 1H), 3.44 – 3.37 (m, 1H), 3.33 (s, 2H), 2.68 (t, J = 7.1 Hz, 1H), 2.49 (t, J = 7.1 Hz, 2H), 2.13 (s, 1H), 1.70 (s, 1H), 1.66 – 1.53 (m, 4H), 1.48 (d, J = 7.7 Hz, 2H). LC MS. Calc for $[M+Na]^+$ 282.4 $C_{11}H_{22}O_6S$, found 282.4

Synthesis of Glc-NC@Man

To a solution of **Glc-NCs** (1 mL, 2.89 mM), the synthesized thio-linked mannose (2.448 mg, 8.67 µmol) was added and let react overnight at room temperature. The **Glc-NC@Man** were dialyzed against 1.5 L of ultrapure water twice.

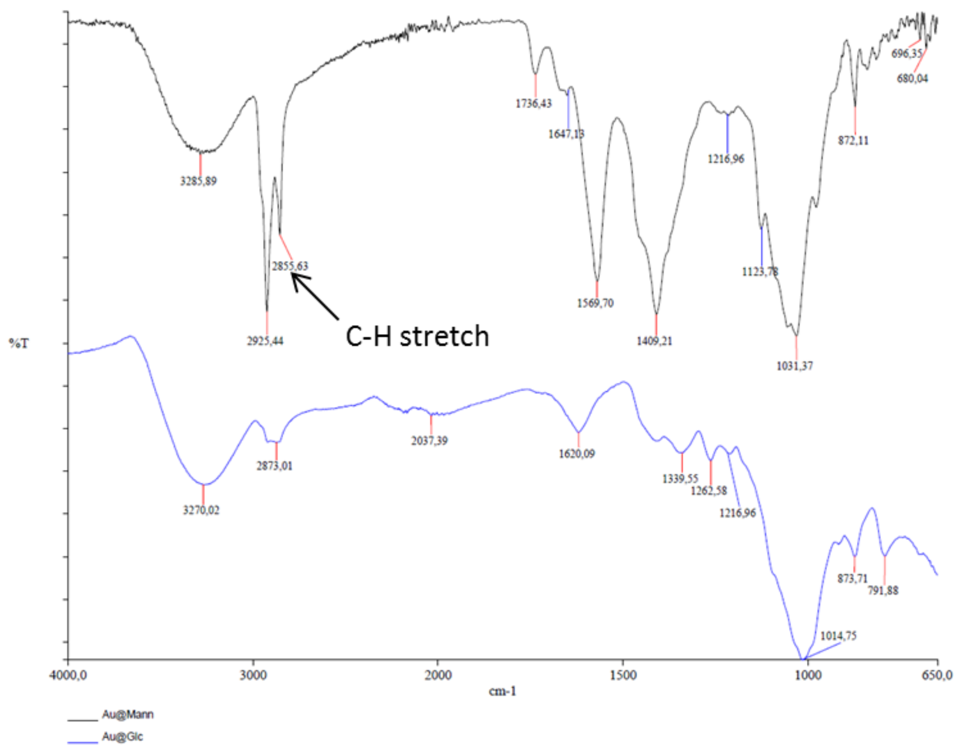

Figure S11: IR spectrum of **Glc-NCs** and **Glc-NC@Man**. The **Glc-NC@Man** contains peaks of the C-H stretch from the linker.

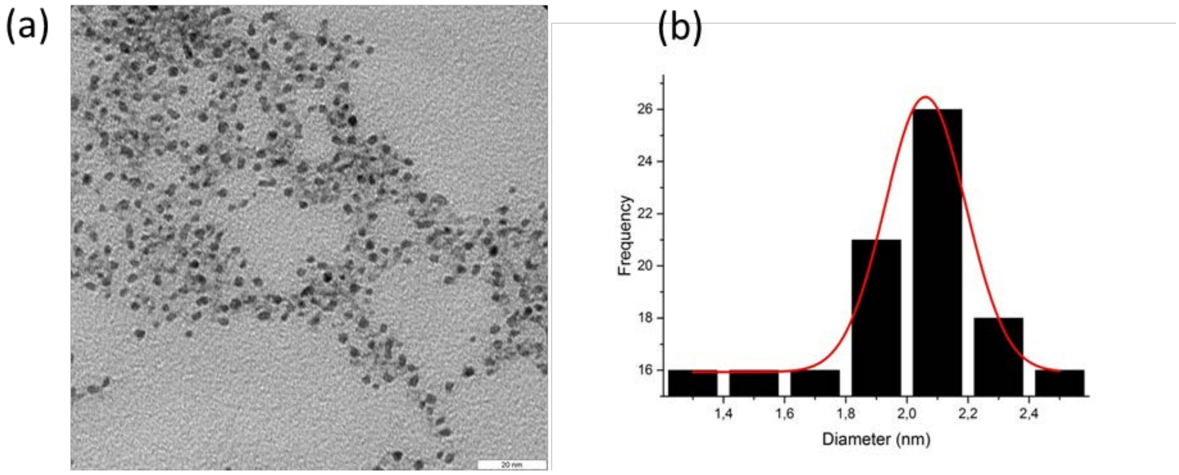

Figure S12: (a) TEM image and (b) size distribution of **Glc-NC@Man** considering 129 nanoclusters. The size of **Glc-NC@Man** was 2.061 ± 0.003 nm.

Biological Studies

Cell culture

The mouse cell line L929 of adipose cells was cultured in 10 cm^2 dishes with RPMI 1640 + 2mM L-Glutamine + 10% Fetal Bovine Serum (FBS) + 0.26μM Cisplatin. The cells were incubated at 37 °C in an ambient air/5% CO_2 atmosphere and sub-confluent cultures (70-80%) were split using DPBS + 0.05% EDTA.

MTS Assay

For the cell viability assessment, the cells were seeded at 13,000 cells/well in 96-well plates and allowed to settle overnight. Then, the cells were incubated with NCs at 500, 100, 50, 25, 12.5, 6.250, 3.125, 1.563, 0.781, 0.391 and 0 mM from a concentrated stock solution of each of the NC samples which was prepared in water and successively diluted in RPMI mixture cell media. For the controls, the same amount of water was added to the most concentrated samples and similarly diluted with the cell medium in order to see the effect of water on the viability of the cells.

After 24 h, the CellTiter 96 A_{Queous} One Solution Cell Proliferation Assay was used. For this assay, 20 μL of MTS [3-(4,5-dimethylthiazol-2-yl)-5-(3-carboxymethoxyphenyl)-2-(4-sulfophenyl)-2H-tetrazolium inner salt] were directly added to the culture wells and incubated for 1-4 hours. The absorbance was then measured at 490 nm using a 96-well plate reader. Untreated cells were assayed as a control reference.

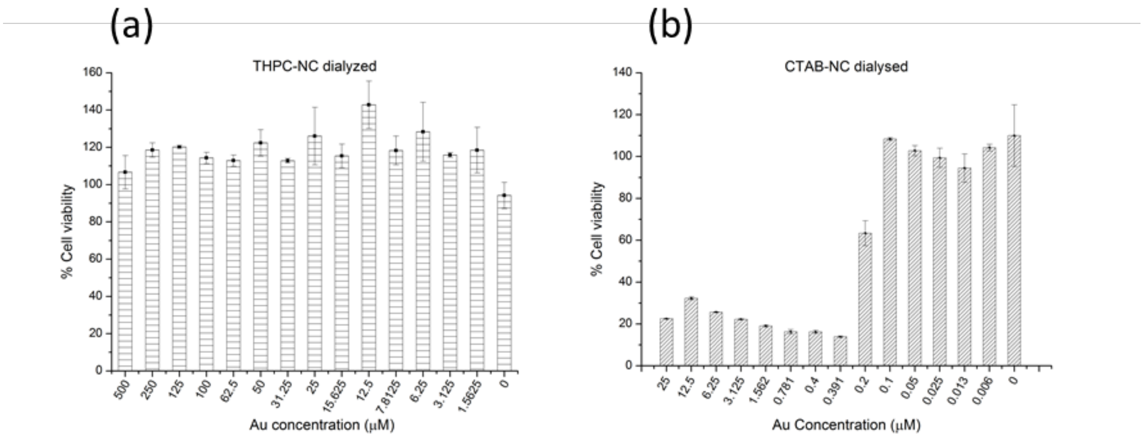

Figure S13: Cytotoxicity of (a) THPC-NCs and (b) CTAB-NCs after three days of dialysis. THPC-NCs did not show toxicity at any of the concentrations studied whereas CTAB-NCs were toxic at higher concentrations than 0.2 µM.

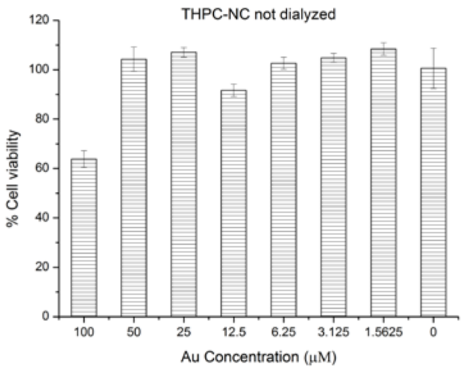

Figure S14: Cytotoxicity of THPC-NCs not dialyzed. THPC-NCs without dialysis showed toxicity at 100 µM.

ICP-OES

For gold uptake measurements, L929 cells were seeded in 10 cm^2 plates. When cells reached confluence in the dish, the cell number was counted (approximately 9 × 10^7 cells per plate) and the **Glc-NCs** added (gold concentrations: 12.5 µM, 25 µM). After a 1 h treatment with **Glc-NCs**, the medium was removed and the cells were washed with DPBS + 0.05% EDTA to collect cell residues. The cell residues were further

digested with 300 μL of HNO_3:HCl (3 : 1) and decomposed for 48 h at room temperature. Subsequently, 700 μL of MilliQ water was added to each tube. Gold concentrations were determined by Inductively Coupled Plasma Optical Emission Spectrometer (ICP – OES) (Optima 8000; Perkin Elmer¸ Massachusetts; USA). An external calibration series from 0.1 mg/L to 5 mg/L was prepared using a gold standard solution.

References

1. Duff, D. G.; Baiker, A.; Edwards, P. P. *Langmuir* **1993**, *9*, 2301.

2. Lin, C.-C.; Yeh, Y.-C.; Yang, C.-Y.;Chen, C.-L.; Chen, G.-F.; Chen, C.-C.; Wu, Y.-C. *J. Am. Chem. Soc.* **2002**, *124*, 3508-3509.

3. Bowers, C. M.; Carlson, D. A.; Rivera, M.; Clark, R. L.; Toone, E. J. *J. Phys. Chem. B* **2013**, *117*, 4755−4762.
